# Mutational analysis of *Plasmodium falciparum* dihydrofolate reductase and dihydropteroate synthase genes in the interior division of Sabah, Malaysia

**DOI:** 10.1186/1475-2875-12-445

**Published:** 2013-12-10

**Authors:** Tiek Ying Lau, Mersumpin Sylvi, Timothy William

**Affiliations:** 1 Biotechnology Research Institute, Universiti Malaysia Sabah, Jalan UMS, 88400 Kota Kinabalu, Sabah, Malaysia; 2 Infectious Disease Department, Queen Elizabeth Hospital, Karung Berkunci No. 2029, Jalan Penampang, 88560 Kota Kinabalu, Sabah, Malaysia

## Abstract

**Background:**

The sulphadoxine/pyrimethamine (SDX/PYR) combination had been chosen to treat uncomplicated falciparum malaria in Malaysia for more than 30 years. Non-silent mutations in dihydrofolate reductase (*dhfr*) and dihydropteroate synthase (*dhps*) genes are responsible for the resistance to pyrimethamine and sulphadoxine, respectively*.* This study reports the mutational analysis of *pfdhfr* and *pfdhps* in single *Plasmodium falciparum* infection isolates from the interior division of Sabah, Malaysian Borneo.

**Methods:**

A total of 22 *P. falciparum* single infection isolates collected from two districts of the interior division of Sabah from February to November 2010 were recruited for the mutational study of *pfdhfr* and *pfdhps*. Both genes were amplified by nested PCR prior to DNA sequencing and mutational analysis.

**Results:**

A total of three *pfdhfr* and four *pfdhps* alleles were identified. The most prevalent *pfdhfr* allele is ANRNL (86%) involving triple mutation at position 108(S to N), 59(C to R) and 164(I to L). In *pfdhps*, two novel alleles, SGTGA (73%) and AAKAA (5%) were identified. Alleles involving triple mutation in both *pfdhfr* (ANRNL) and *pfdhps* (SGTGA), which were absent in Sabah in a study conducted about 15 years ago, are now prevalent.

**Conclusions:**

High prevalence of mutations in SDX/PYR associated drug resistance genes are reported in this study. This mutational study of *pfdhps* and *pfdhfr* indicating that SDX/PYR should be discontinued in this region.

## Background

*Plasmodium falciparum*, which causes the most severe form of malaria, is found mainly in the tropical and subtropical regions including Sabah, Malaysian Borneo. The spread of resistance to anti-malarial drugs poses an important health problem. Chloroquine-resistant *P. falciparum* was first reported in Malaysia in 1966 and the sulphadoxine-pyrimethamine (SDX/PYR) (Fansidar®) combination replaced chloroquine in 1979 as first-line treatment for uncomplicated falciparum malaria in Malaysia. The combination of pyrimethamine and sulphadoxine gives a synergistic action against *P. falciparum* by inhibiting the dihydrofolate reductase and dihydropteroate synthase enzymes in the folate biosynthesis pathway, respectively [1,2]. Mutations in the genes encoding for dihydrofolate reductase (*dhfr*) and dihydropteroate synthase (*dhps*) are associated with resistance to SDX/PYR [3].

In Malaysia, artemisinin combination therapy (ACT) is currently being used as the first-line therapy for falciparum malaria. ACT in Sabah consists of either arthemether/lumefantrine or artesunate/mefloquine. This is the first choice for the treatment of uncomplicated *P. falciparum* infections in Sabah according to the State treatment policy guidelines. However, despite this policy, SDX/PYR combination is still occasionally used by field workers to treat *P. falciparum* as presumptive treatment for patients with suspected malaria who live deep in the interior where a timely microscopy result for malaria parasites may not be possible. Besides, SDX/PYR combination is also sometimes used for malaria prophylaxis in this region.

Molecular techniques based on the detection of mutations in parasite molecules targeted by anti-malarial drugs may offer better tools for monitoring drug resistance. *In vitro* resistance of *P. falciparum* to PYR is primarily conferred by a non-synonymous point mutation at S108N and is progressively enhanced by mutations at residue N51I and/or C59R of *pfdhfr*. An additional mutation at I164L is associated with high-level clinical resistance to SDX/PYR [4,5]. In *pfdhps*, point mutations at A437G and K540E are considered responsible for SDX resistance. It was thought that the point mutation at 437 is the first event and is associated with a decreased response to SDX [3,6]. Mutations at A581G and A613S in the background of A437G were shown to associate with high clinical resistance to SDX/PYR in South America and Southeast Asia [5,7].

A large-scale study on the clinical efficacy of *P. falciparum* malaria to SDX/PYR in Peninsular Malaysia conducted in mid 1990s, reported 47% of the study isolates were resistant to SDX/PYR [8]. A subsequent molecular study reported 87% of *P. falciparum* had triple mutations in *pfdhfr* and all isolates having point mutation at codon A437G of *pfdhps* as indication of drug pressure, accompanied by 81% point mutation at codon A581G indicating reduced *in vitro* responsiveness of SDX [4]. The authors speculated that SDX/PYR might be still effective in treating uncomplicated *P. falciparum* malaria in Sabah due to most of the isolates having two or less *dhfr* mutations. An increasing number of imported cases of malaria as a result of worldwide travel and the use of migrant work forces to and from the neighbouring endemic countries have been reported in Sabah. This makes Sabah vulnerable to the introduction of drug resistant malaria and a reassessment of molecular markers associated with *P. falciparum* resistance would be timely. In this study, point mutations at *pfdhfr* and *pfdhps* genes in *P. falciparum* isolates from the Interior Division of Sabah, Malaysian Borneo were typed.

## Methods

### Study sites and sample collections

A total of 22 *P. falciparum* single infection samples out of 243 total samples collected from the districts of Keningau, and Nabawan in the interior division of Sabah, Malaysian Borneo from February to November in 2010, were analyzed in this study. Ethical clearance was obtained from the Ethical Committee of Ministry of Health Malaysia and the Ethical Committee of Universiti Malaysia Sabah prior Blood samples of approximately 25 μl were collected on chromatography paper (Whatman 3MM) and dried before storage in an individual plastic bag for sample collection. Patients’ verbal and written consent were obtained before their blood was taken. Sample collection and microscopic examination was carried out by qualified medical laboratory staff at the respective hospitals.

### Detection of mutations in *pfdhfr* and *pfdhps*

Genomic DNA was extracted using QIAmp® DNA Mini Kit (QIAGEN, Hilden, Germany), according to the manufacturer’s recommendation. *Plasmodium* species were identified by nested-PCR using genus and species-specific primers based on the small subunit ribosomal RNA gene (*ssrRNA*). Single *P.falciparum* infections were recruited in this study.

*pfdhfr* was amplified in nested PCR according to previously described method with minor modifications [9]. Primary round amplification comprised of 4 μl DNA template, 0.25 μM each primers, 1.5 mM MgCl_2_, 200 μM dNTPs, 1× PCR buffer, and 2.5 U of Taq in a 20 μl reactions. Cycling conditions were performed as follow; 94°C for 3 min, followed by 45 cycles of 94°C for 30 sec, 45°C for 45 sec, and 72°C for 45 sec and finally 72°C for 5 min. Nested PCR was performed with 2 μl DNA template from the first round PCR product, 0.25 μM primers, 1.5 mM MgCl_2_, 200 μM dNTPs, 1× PCR buffer, and 1 U of Taq in a 20 μl reactions. The PCR was carried at 94°C for 3 min, followed by 40 cycles at 94°C for 1 min, 45°C for 1 min, 72°C for 1 min and finally 72°C for 10 min.

Amplification of *pfdhps* was performed in nested PCR according to previously described method with minor modifications [10]. First round of PCR was carried out in a 20 μl reaction containing 4 μl template, 0.25 μM primers, 2.5 mM MgCl_2_, 200 μM dNTPs, 1× PCR buffer, and 2.5 U of Taq at the following PCR conditions, 94°C for 5 min, followed by 30 cycles 94°C for 55 sec, 45°C for 45 sec, 72°C for 45 sec and finally 72°C for 5 min. Nested PCR comprising 2 μl DNA template from the first round PCR product, 0.25 μM primers, 1.5 mM MgCl_2_, 200 μM dNTPs, 1× PCR buffer, and 1.0 U of Taq in a 20 μl reaction. PCR was carried out at 94°C for 3 min, followed by 40 cycles at 94°C for 1 min, 45°C for 1 min, 72°C for 1 min and 72°C for 10 min. Primer sequences for nested PCR amplification of *pfdhps* and *pfdhfr* is shown in Table 1.

**Table 1:** **Primer sequence for the amplification of *pfdhfr *** **and *pfdhps***

**PCR reaction**		**Primer pair sequence**	**Expected size**	**Reference**
*pfdhfr*	Nest 1	Pfdhfr_D1 5′ TTTATATTTTCTCCTTTTTA 3′	718 bp	[9]
		Pfdhfr_D2 5′ CATTTTATTATTCGTTTTCT 3′		
	Nest 2	Pfdhfr_M1 5′ TTTATGATGGAACAAGTCTGC 3′	648 bp	
		Pfdhfr_M5 5′ AGTATATACATCGCTAACAGA 3′		
*pfdhps*	Nest 1	Pfdhps_N185 5′ TGATACCCGAATATAAGCATAATG 3′	1031 bp	[10]
		Pfdhps_N218 5′ ATAATAGCTGTAGGAAGCAATTG 3′		
	Nest 2	Pfdhps_Rc 5′ GGTATTTTTGTTGAACCTAAACG 3′	728 bp	
		Pfdhfr_D2 5′ ATCCAATTGTGTGATTTGTCCAC 3′		

PCR products were purified using QIAquick Gel Extraction Kit (QIAGEN, Hilden, Germany) prior to DNA sequencing. Detection of the mutations was performed using the MEGA version 5 through comparative analysis with the wild type sequence (GenBank accession numbers are XM_001351443 and Z30654 for *pfdhfr* and *pfdhps*, respectively). Single nucleotide polymorphisms were verified through the bidirectional sequencing.

## Results

### Mutational analysis of *pfdhfr*

Based on the analysis of 5-codon alleles in *pfdhfr,* the wild type allele*,* ANCSI was absent in the 22 *P. falciparum* isolates in the interior division of Sabah. Three different alleles, namely ANRNL, ANRNI and AIRNI were detected (Table 2a). The most prevalent allele in this study is ANRNL (86.4%), which was absent in previous study conducted in Sabah about 15 years ago [4]. All isolates had mutation at position S108N and C59R accompanied by 86.4% (n = 19) mutation at I164L. Approximately 9.1% (n = 2) of the total isolates showing *pfdhfr* double mutations at position S108N and C59R alone (ANRNI). One isolate from this region had additional mutation at N51I, which has never been reported in Sabah, giving rise to allele AIRNI. This allele was prevalent in *P. falciparum* isolates from Kelantan, Peninsular Malaysia where clinical resistance to SDX/PYR has been reported in various locations [4].

**Table 2:** **Dihydrofolate reductase ( *dhfr *) and dihydropteroate synthase ( *dhps *) alleles in *Plasmodium falciparum *** **isolates from the interior division of Sabah, Malaysian Borneo**

**(a)**	***pfdhfr *** **amino acid position**					**Prevalence (%)**
	16	51	59	108	164	n = 22
	A	N	*R*	*N*	*L*	86.4
	A	*I*	*R*	*N*	I	4.5
	A	N	R	N	I	9.1
**(b)**	***pfdhps *** **amino acid position**					**Prevalence (%)**
	436	437	540	581	613	n = 22
	S	*G*	*T*	*G*	A	72.7
	*A*	A	K	A	A	4.5
	S	*G*	K	A	A	9.1
	S	G	K	G	A	13.6

*Single letter amino acid denoting the wild type and point mutations are indicated with italics. The wild type allele of *pfdhfr* is ANCSI and *pfdhps* is SAKAA.

### Mutational analysis of *pfdhps*

Four alleles of *pfdhps* namely SGTGA, SGKGA, SGKAA and AAKAA but not the wild type allele SAKAA, were identified in this study (Table 2b). SGTGA is the most prevalent allele which present in 72.7% (n = 16) of the total study isolates. Only 13.6% (n = 3) of the *P. falciparum* isolates had the SGKGA allele, which was previously the most common allele (76%) in Sabah [4]. A novel point mutation at position 540 from lysine (K) to threonine (T), instead of glutamic acid (E) was prevalent in Sabah (72.7%). K540T point mutation is unique to *P. falciparum* isolates in Sabah. It is noteworthy that K540E was reported in Sabah in the previous study but was absent in this study. Besides, one isolate had mutation at S436A, which is also novel to Sabah *P. falciparum* isolates.

## Discussion

Single or multiple mutations at residues A16V, N51I, C59R, S108N and I164L of *pfdhfr* have been reported to be associated with resistance to antifolate drugs in falciparum malaria treatment. Of these, mutations at N51I, C59R, S108N and I164L were shown to correlate with resistance to pyrimethamine [5,11]. Mutation at A16V was specifically associated with cycloguanil resistance [12]. Mutation at S108N was thought to be the first event which resulted in reduce *in vitro* responsiveness to pyrimethamine, followed by point mutation at N51I and/or C59R and finally at position I164L. A previous report implicated that an accumulation of mutations were associated with increasing resistance to pyrimethamine [11]. In this study, 86.4% of the *P. falciparum* isolates had triple mutations at C59R, S108N and I164L (ACNRNL). In accordance with the previous study, all of the isolates showed mutation S108N as expected in populations where SDX/PYR has been used extensively. However, in contrast to the previous study that showed 2% prevalence of wild type allele at codon 59 [5], there was no wild type allele at this position in the current study.

Triple mutations involving the residue I164L were commonly found in areas with high levels of clinical resistance to SDX/PYR [5]. It is worth noting that even though mutation at A16V was not detected in the present study, the presence of triple mutation at C59R, S108N and I164L could provide cross-resistance to cycloguanil [13,14]. Cycloguanil is the active metabolite of proguanil, an antifolate drug that is commonly used in the prophylaxis of *P. falciparum* malaria. Prophylactic use of proguanil was widespread in Malaya in the early 1950s and it is used in combination with atovaquone as prophylaxis by travellers to Malaysia.

A previous study showed that double mutation at C59R and S108N was prevalent in Sabah and predicted not to cause clinical resistance to pyrimethamine [4]. Recently, studies conducted in Angola and Iran also reported that double mutation at residues C59R and S108N are more likely to act as a predictor for the development of resistance to antifolate drugs [15,16]. Taking this into consideration, additional mutation at residue I164L found in this study at high prevalence could be the pivotal mutation that causes clinical resistance of *P. falciparum* to SDX/PYR therapy in Sabah. Previous report showed that the presence of three *dhfr* point mutations were correlated with clinical resistance to SDX/PYR, regardless of *dhps* alleles [4].

In *pfdhps*, mutation at position A437G is associated with sulphonamide resistance, while additional point mutations at S436A, K540E, A581G and A613S might increase the degree of resistance. *Plasmodium falciparum*, harbouring double mutations at residues A437G and K540E (SGEAA), were known to be associated with resistance to sulphadoxine [17,18]. In Sabah, allele SGKGA was predominant ten years ago, while the current finding showed only approximately 14% (3/22) allele SGKGA. Additional mutation at K540T that gave rise to allele SGTGA was the most prevalent allele (72.7%). Mutation at K540T is unique to the *P. falciparum* isolates from Sabah, as amino acid change from lysine to glutamic acid (K540E) was commonly found. High prevalence of K540T in *P. falciparum* isolates in this region due to the nonsynonymous amino acid substitution (AAA → ACA) instead of K540E (AAA → GAA) need to be highlighted. *Plasmodium falciparum* isolates carrying 540 T have replaced isolates with 540E within the timeframe of a decade. High prevalence of 540 T and absence of 540E indicating that this codon has high tendency subjecting to natural selection and might enhance the drug resistance pressure of *P. falciparum* carrying this mutation. Besides, recent studies reported a change from lysine to asparagine (K540N) at a few geographical locations, indicating mutations at codon 540 are more likely to change to different amino acids [19,20]. In this study, mutation at K540T occurred as a result of non-synonymous mutation at nucleotides coding for lysine (AAA) to threonine (ACA). Triple mutations at residues S436A, K540T and A581G (SGTGA) could contribute to the resistance to sulphadoxine in Sabah, thus decreasing the efficacy of SDX/PYR. One novel allele (AAKAA) involving mutation at residue S436A was found in one of the isolates and is not thought to cause resistance to sulphadoxine. This allele is sometime referred as wild type allele in a previous report [21].

A four-allele combination of *pfdhfr-pfdhps*, namely ANRNI-SGKAA, AIRNI-AAKAA, ANRNL-SGKGA and ANRNL-SGTGA were formed in this study (Table 3). The sextuple mutations (ANRNL-SGTGA), which comprise triple mutations in both genes, are most prevalent (72.7%) in Sabah. Overall, the present study reported a progressive mutation in *dhfr* and *dhps* of *P. falciparum* isolates in the interior division of Sabah, where triple mutations are now prevalent in both genes compared to the previous study with double mutations on both genes. A sextuple allele combination of *pfdhfr-pfdhps* is associated with high-grade clinical resistance to SDX/PYR, which was found mainly in Africa [22,23]. The other three alleles found in Sabah range from triple mutation to quintuple mutations (ANRNI-SGKAA, AIRNI-AAKAA, ANRNL-SGKGA), which could modulate varying degree of resistance to SDX/PYR therapy.

**Table 3:** **Allele combinations of *pfdhfr *** **and *pfdhps *** **in *Plasmodium falciparum *** **isolates from the interior division of Sabah, Malaysian Borneo**

***Pfdhfr-pfdhps *** **allele combinations**	**Prevalence (%)**
AN***RNL***-S***GTG***A	72.7
AN***RNL***-S***G***K***G***A	13.6
A***IRN***I-***A***AKAA	4.5
AN***RN***I-S***G***KAA	9.1

*Single letter amino acid denoting the wild type and point mutations are indicated with italics.

## Conclusions

This preliminary study showed that mutations in *pfdhfr* and *pfdhps,* which are associated with SDX/PYR resistance are now prevalent in Sabah. The spread of resistant *P. falciparum* to SDX/PYR in Sabah making this drug combination is no longer effective in combating *P. falciparum* in this geographical region. This report also confirms that treatment with this combination is unlikely to be effective and should thus be discontinued. Artemisinin-based combination therapy (artesunate/mefloquine or artemether/lumefantrine) should continue to be used as the preferred treatment for falciparum malaria. Further study is required to elucidate the close relationship of these mutations with the susceptibility of *P. falciparum* isolates to SDX/PYR in Sabah.

## Competing interests

The authors declare that they have no competing interests.

## Authors’ contributions

TYL conceived and designed the study. WT was responsible for coordinating sample collection. MS performed the experiments and data analysis. TYL MS and WT participated in manuscript preparation. All authors read and approved the final manuscript.
